# Myopia: a serious condition that needs our attention

**Published:** 2019-05-13

**Authors:** Hasan Minto, Priya Morjaria, Kovin Naidoo

**Affiliations:** 1 Global Programs Director: Child Eye Heath & Low Vision Brien Holden Vision Institute.; 2 Research Fellow and Public Health Optometrist: London School of Hygiene & Tropical Medicine.; 3 Senior Vice President: Inclusive Business, Philanthropy & Social Impact, Essilor.

**Myopia is a growing epidemic that will affect half the global population by 2050, and its complications can cause irreversible visual loss.**

**Figure:**
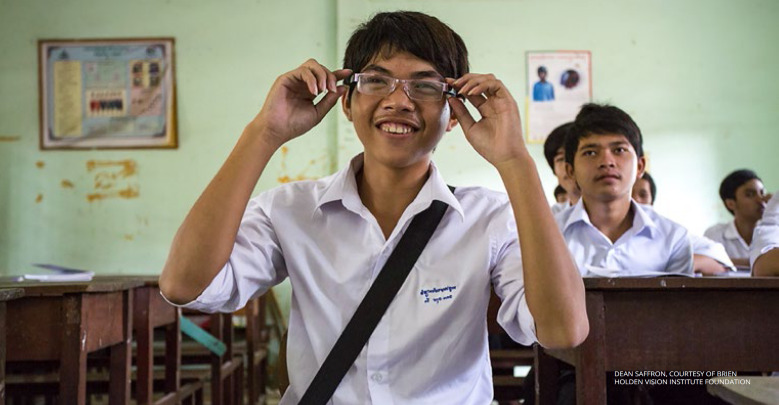
Although a simple pair of spectacles will correct visual loss, they cannot prevent the blinding consequences of high myopia. CAMBODIA

People with myopia – or short-sightedness – are unable to see objects that are far away, but can see near objects clearly. This affects every aspect of their life, including education, employment and safety.

Over 2 billion people worldwide are estimated to have myopia, defined as ≤ −0.5 dioptres (D). Of these, around 10% have high myopia, defined as ≤ −5 D.^1^,^2^ People with high myopia are at increased risk of potentially blinding eye conditions such as macular degeneration, retinal detachment, open-angle glaucoma and cataract.

Myopia is already a major public health challenge. In 2015, an estimated 480 million people worldwide were considered blind or visually impaired because they did not have access to spectacles,^3^ making myopia the leading cause of visual impairment and blindness worldwide.

By 2050,^2^ myopia is expected to affect 5 billion people, which is half of the projected global population at that time. This will place an even greater burden on health services to provide spectacles and to prevent and manage the conditions associated with high myopia.

Uncorrected myopia, together with macular degeneration, were estimated to be responsible for a US $250 billion loss in global productivity in 2015.^4^ As myopia becomes more common, this is set to rise.

## Making a difference

Meeting the need for myopia correction, and slowing down or reversing the global increase in myopia, requires that we address service delivery, access to affordable correction, health promotion, advocacy and policy change at both national and global level. Collaboration between government, civil society, researchers, innovators and the private sector – rather than competition – is essential. The International Myopia Institute (**www.myopiainstitute.org**) is one example of a collaboration that is bringing about consensus on key issues such as definitions, clinical guidelines, clinical trials, and how to involve industry. We need more coalitions around education, service delivery, health promotion, advocacy, and research. Coalitions and partnerships will allow us to scale up efforts and make the impact that is needed.

**Governments** must take the lead in addressing the increase in myopia. National policies must address child eye health specifically; e.g., by making eye examinations compulsory for children at school entry and making it easier to import (or manufacture) products and drugs that can help to control myopia progression. Healthy school initiatives should include spending time outdoors as this has been shown to delay the onset of myopia (which means that children would be less likely to develop high myopia). Schools provide an ideal point of contact between health and refractive error services for children and their parents; e.g., by hosting health promotion activities that encourage parents to take children for an eye examination and to get the appropriate correction for them.

**The private sector** must support all components of a comprehensive approach, be it service delivery, human resource development, advocacy, policy change, research or health promotion. Industry should drive the agenda to create advanced, yet affordable, myopia control products, whether contact lenses or spectacle lenses, and make them accessible for all.

**Non-governmental organisations** (NGOs) involved in eye health are key to supporting the comprehensive approach by prioritising advocacy and policy change. NGOs must support the scaling up of services rather than see themselves as a replacement for either government or practitioners. They play a crucial role and can adopt a more active approach to influencing change. It is vital that myopia is included in World Health Organization, UNICEF, and other broader development agendas, as myopia has the potential to slow the education of our children and thus hamper efforts to achieve the United Nations' Sustainable Development Goals (**www.un.org/sustainabledevelopment**).

**Figure 1:**
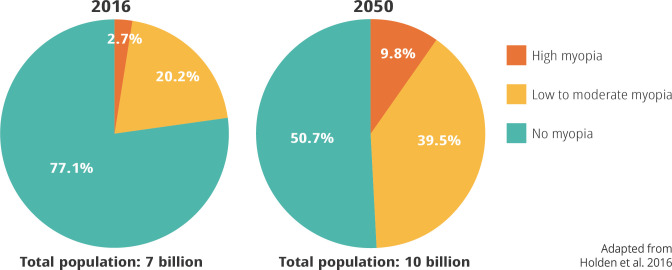
Myopia and high myopia are rapidly increasing worldwide

**Optometrists, ophthalmologists and allied eye health professionals** can all play a role in reducing the detrimental impact on quality of life due to myopia. Health promotion and education need to become a critical component of patient management. Eye care professionals will also need to support efforts to change government policy and use their connection in the community to become advocates for these changes. This issue discusses evidence for the myopia epidemic and the risks of high myopia along with the interventions available to reduce the risk of myopia and slow down its progression. While there are clinical and optical interventions to slow down the progression of myopia, lifestyle and environmental changes (less near work and more time outside) are arguably of greater importance as it protects children against the onset of myopia. We have included practical tips on how to detect, refer, diagnose and manage myopia. A list of data that are required for monitoring myopia management are described to encourage clinicians to begin monitoring myopia progression in patients.

**School eye health**

**Figure:**
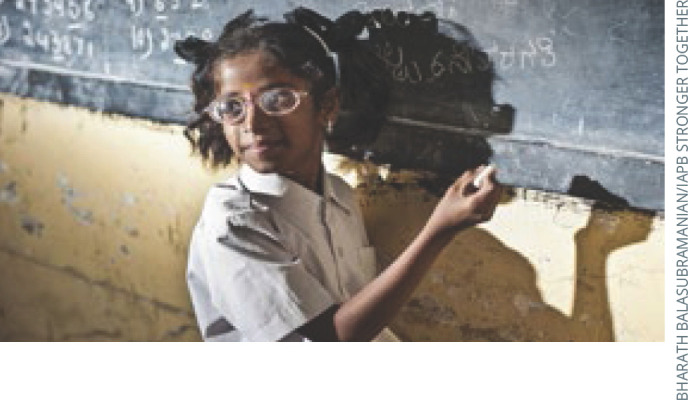
School eye health programmes form an integral part of the global effort to address myopia. Read our 2017 issue on School Eye Health here: **www.cehjournal.org/school-eye-health**
